# Silver Nanofunctionalized Stent after Radiofrequency Ablation Suppresses Tissue Hyperplasia and Bacterial Growth

**DOI:** 10.3390/pharmaceutics14020412

**Published:** 2022-02-14

**Authors:** Yubeen Park, Dong-Sung Won, Ga-Hyun Bae, Dae Sung Ryu, Jeon Min Kang, Ji Won Kim, Song Hee Kim, Chu Hui Zeng, Wooram Park, Sang Soo Lee, Jung-Hoon Park

**Affiliations:** 1 Biomedical Engineering Research Center, Asan Medical Center, Asan Institute for Life Science, 88 Olympic-ro 43-gil, Songpa-gu, Seoul 05505, Korea; ybeen9733@amc.seoul.kr (Y.P.); ehdtjd3004@amc.seoul.kr (D.-S.W.); kryuds@amc.seoul.kr (D.S.R.); miny2208@amc.seoul.kr (J.M.K.); kimjw203@amc.seoul.kr (J.W.K.); zxcuiop55@amc.seoul.kr (S.H.K.); angelazeng29@amc.seoul.kr (C.H.Z.); 2 Asan Medical Center, Department of Gastroenterology, University of Ulsan College of Medicine, 88 Olympic-ro 43-gil, Songpa-gu, Seoul 05505, Korea; 3 Department of Biomedical-Chemical Engineering, The Catholic University of Korea, 43 Jibong-ro, Bucheon 14662, Korea; ckd016@catholic.ac.kr (G.-H.B.); wrpark@catholic.ac.kr (W.P.); 4 Department of Integrative Biotechnology, College of Biotechnology and Bioengineering, Sungkyunkwan University, 2066 Seobu-ro, Jangan-gu, Suwon 16419, Korea

**Keywords:** malignant biliary obstruction (MBO), radiofrequency (RF) ablation, silver nanoparticle (AgNP), self-expandable metal stent (SEMS), tissue hyperplasia, antibacterial effect

## Abstract

Intraductal radiofrequency (RF) ablation combined with placement of a self-expandable metal stent (SEMS) for malignant biliary obstruction has risks such as stent- and heat-induced biliary sludge and restenosis. Here, we investigated the efficacy of a silver nanoparticles (AgNPs)-coated SEMS to inhibit tissue hyperplasia and bacterial growth caused by RF ablation with stent placement in the rabbit bile duct. The release behavior and antibacterial effects of AgNPs-coated SEMSs were evaluated. Then, SEMSs were successfully placed in all rabbits immediately after RF ablation. Ag ions were rapidly released at the beginning and then showed a gradual release behavior. The AgNPs-coated SEMS significantly inhibited bacterial activity compared to the uncoated SEMS (*p* < 0.05). Cholangiography and histological examination confirmed that the level of tissue hyperplasia was significantly lower in the AgNPs group than in the control group (all *p* < 0.05). Immunohistochemistry analyses revealed that TUNEL-, HSP 70-, and α-SMA-positive areas were significantly lower in the AgNPs group than in the control group (all *p* < 0.05). Intraductal RF ablation combined with nanofunctionalized stent placement represents a promising new approach for suppressing thermal damage as well as stent-induced tissue hyperplasia and bacterial growth.

## 1. Introduction

Malignant biliary obstruction (MBO) is caused by various factors, including cholangiocarcinoma, pancreatic cancer, extrinsic compression, and adjacent inflammation. As MBO is often diagnosed at a later stage, the proportion of patients who undergo surgical resection is limited to only 30%, resulting in a high mortality rate [1,2,3,4]. Placement of a self-expandable metal stent (SEMS) is the current standard treatment for palliation in patients with unresectable MBO [5,6,7,8]. However, SEMSs are susceptible to recurrent symptoms due to tumor ingrowth, tissue hyperplasia, biofilm deposition, and biliary sludge formation with restenosis, which occur within 6 to 8 months [5,8,9,10]. Intraductal radiofrequency (RF) ablation has been presented as a novel option for the palliative management of unresectable MBO [4,11,12,13]. RF ablation generates high-frequency alternating currents to create localized heating and induce coagulative necrosis in tumor tissues, thus maintaining patency and optimal drainage in the biliary tract. RF ablation is easy to perform, and its safety and efficacy have been verified through various clinical trials [12,13,14,15,16]. However, thermal damage to the bile duct may also lead to health tissues, and stenosis may occur owing to edema, swelling, inflammation, and cicatrization [11,17]. Consequently, RF ablation should be used in combination with SEMS placement; the combination of RF ablation and SEMS has shown better clinical outcomes in terms of biliary tract patency than SEMS placement or RF ablation alone [18,19]. However, this combination is associated with bacterial colonization, which can increase the risk of inflammatory response and sepsis, leading to cholangitis, pancreatitis, cholecystitis, and gallbladder empyema [19,20,21,22].

**Figure 1:**
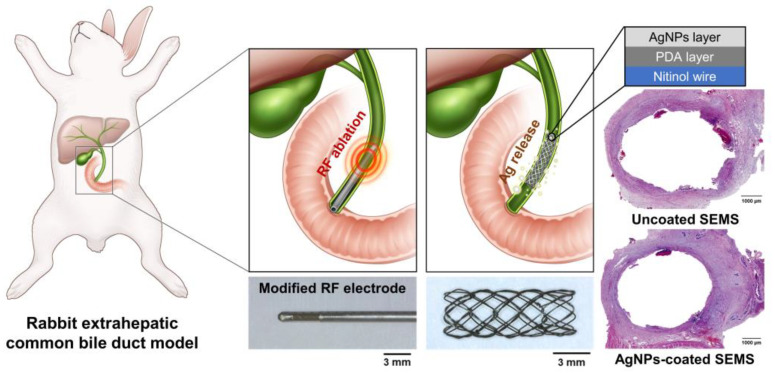
Schematic illustration of silver nanoparticles (AgNPs)-coated self-expandable metal stent (SEMS) placement immediately after radiofrequency (RF) ablation in a rabbit extrahepatic common bile duct model. AgNPs-coated SEMS suppressed bacterial growth and tissue hyperplasia due to inflammatory responses caused by local heating and stent placement.

Silver nanoparticles (AgNPs) have antibacterial, anti-inflammatory, antiviral, antioxidant, anti-angiogenesis, and antiplatelet properties and have been used for the prevention of infection in various clinical fields [23,24,25]. Stents coated with AgNPs of various sizes have been actively investigated to prevent in-stent restenosis caused by tissue hyperplasia along with inflammatory responses and biofilm formation resulting from bacterial adherence [10,26,27,28]. In a previous study, AgNPs-functionalized SEMSs exerting antibacterial and anti-inflammatory effects were introduced to suppress tissue hyperplasia adjacent to the stented rat esophagus and rabbit bile duct [10,26]. Functionalized stents with surface modifications using various nanoparticles represent a promising new strategy to overcome stent-related restenosis and to provide controlled local treatment [10,26,27,28,29,30,31]. We hypothesized that placement of an AgNPs-coated SEMS immediately after RF ablation could inhibit thermal damage as well as stent-induced bacterial and inflammatory responses (Figure 1). Therefore, the purpose of this study was to investigate the efficacy of AgNPs-coated SEMS placement immediately after RF ablation in suppressing thermal damage as well as SEMS-induced tissue hyperplasia and bacterial growth in the extrahepatic common bile duct of rabbits.

## 2. Materials and Methods

### 2.1. Preparation and Surface Characteristics of AgNPs-Coated SEMS

All stents were knitted from a single thread of 0.110 mm thickness nitinol wire. The stents were 4 mm in diameter and 10 mm in length. AgNPs-coated stents were prepared as described previously [10]. In brief, SEMS was primarily surface-modified with polydopamine. Then, AgNPs with a size of 20 to 500 nm were attached to the surface-modified SEMS using a silver nitrate (AgNO_3_; Sigma-Aldrich, Burlington, MA, USA) solution with a concentration of 12 mg/mL. The surface characteristics were analyzed by field emission scanning electron microscopy (MAIA3; TESCAN Inc., Brno, Czech Republic) with energy-dispersive X-ray spectroscopy (X-MaxN; Oxford Instruments, Abingdon, UK). Both uncoated and AgNPs-coated SEMSs were subjected to elemental analysis of Ag and surface morphology analysis.

### 2.2. Release of Ag Ions from AgNPs-Coated SEMS

AgNPs-coated SEMSs were immersed in phosphate buffer saline (PBS, 1X, pH 7.4; Thermo Fisher Scientific, Waltham, MA, USA) and incubated for 7 days at 37 °C and 200 rpm in a shaking incubator. Pretreatment of the release solution included mixing it with a 70% nitric acid solution. At a predefined time point, the concentration of Ag ions released into PBS was evaluated using inductively coupled plasma-mass spectroscopy (iCAP Q; Thermo Fisher Scientific).

### 2.3. Bacterial Resistance of AgNPs-Coated SEMS

Antibacterial activity was assayed using *Escherichia coli* (DH5α; Real Biotech Corporation, Taipei, Taiwan), which is known as the main causative agent of cholangitis [32,33]. Briefly, a single colony of *E. coli* cultured on solid Luria-Bertani (LB) agar (LB Broth, Lennox; NEOGEN, Lansing, MI, USA) was inoculated in 3 mL of LB liquid broth and then incubated at 37 °C for 12 h. Next, 200 μL of the cultured bacterial suspension was collected and incubated with uncoated (*n* = 3) and AgNPs-coated (*n* = 3) SEMSs, respectively, in fresh LB broth for 24 h and monitored. At each time point (0, 1, 2, 4, 6, 18, and 24 h), the suspension was transferred to a 96-well plate, and absorbance was measured at 600 nm using a microplate reader (Synergy H1; BioTek, Winooski, VT, USA) to confirm the bacterial inhibitory effect of Ag ions.

In addition, the uncoated SEMS and AgNPs-coated stent were immersed in PBS and incubated at 37 °C for 7 days to evaluate the long-term antibacterial activity. On days 0, 1, 2, 3, and 7, PBS immersing the stent was sampled, and the stent was transferred to fresh PBS. The sampled PBS (200 μL) was then mixed with the *E. coli* suspension (10 μL), and the mixture was incubated at 37 °C for 12 h. Next, the mixture was spread on LB agar, incubated for 18 h, and photographed to confirm the growth of *E. coli* colonies.

### 2.4. RF Ablation Equipment and Electrode

A CoATherm AK-F200 RF generator (APRO KOREA, Gunpo, Korea) and an F180707 RF electrode (APRO KOREA) were used for this experiment. The RF electrode was an 18 G needle-type electrode of 70 mm in length. A 4 mm electrode made of platinum was attached to the distal portion of the needle. To prevent injury to the bile duct during insertion of the needle-type electrode, the distal end of the needle tip was grinded to a tapered shape (Figure S1, Supplementary Materials).

### 2.5. Animal Study Design

This study was approved by the Institutional Animal Care and Use Committee of Asan Institute for Life Sciences (IACUC no. 2020-12-148, Asan Medical Center, Seoul, Korea) and it conformed to the United States National Institutes of Health guidelines for humane handling of laboratory animals. A total of 16 male New Zealand White rabbits (weight range, 2.8–3.4 kg; JA BIO, Suwon, Korea) were used in this study. Four rabbits received RF ablation for determination of the optimal ablation time. The other 12 rabbits were randomly divided into two groups for verification of the efficacy of AgNPs-coated SEMS placement immediately after RF ablation: the control group (*n* = 6) received an uncoated SEMS, and the AgNPs group (*n* = 6) received an AgNPs-coated SEMS. All rabbits were supplied with food and water *ad libitum* and maintained at a mean temperature (±standard deviation) of 24 °C ± 2, with a 12 h day–night cycle.

### 2.6. Protocol for RF Ablation in the Rabbit Bile Duct

RF ablation was performed at 480 kHz and 10 W, and the temperature was controlled within 70 °C. The four rabbits received RF ablation for 40, 60, 90, and 120 s, respectively. The temperature changes and thermal images at the ablation sites were examined by a thermal camera (A400; FLIR Systems, Wilsonville, OR, USA) during the procedure. Cholangiography, gross examination, histological analysis, and immunohistochemistry (IHC) analysis were performed to evaluate the perforation or degree of thermal injuries in the bile duct. All rabbits were immediately sacrificed after the procedure.

### 2.7. RF Ablation with Stent Placement

Anesthesia was induced by intramuscular injection using a mixture of 50 mg/kg zolazepam and 50 mg/kg tiletamine (Zoletil; Virbac, Nice, France) and 5 mg/kg xylazine (Rompun; Bayer HealthCare, Leverkusen, Germany). Moreover, anesthesia was maintained by inhalation of 2% isoflurane (Ifran^®^; Hana Pharm. Co., Seoul, Korea) with 1:1 oxygen (510 mL/kg per min).

The rabbit was placed in supine position, and a 3 cm midline incision was made on the epigastrium. The gastroduodenal junction connecting the distal stomach and proximal duodenum was gently turned inside out, exposing the ampulla of Vater (Figure S2a). The duodenum was then punctured within 1 cm of the ampulla of Vater using a 16 G angiocatheter with a needle (BD Angiocath Plus; Becton Dickinson, Franklin Lakes, NJ, USA), avoiding the vessels. After penetrating the duodenal wall, the needle was removed while holding the Teflon sheath in place. A sheath tip was manipulated into the orifice of the ampulla of Vater, and cholangiography was performed to assess the bile duct. After cholangiography, the RF electrode was inserted through the sheath into the common bile duct, and the center of the electrode was positioned 2.5 cm from the puncture site (Figure S2b). RF ablation was performed for 60 s at 70 °C, after which the RF electrode was removed from the bile duct. An 18 G angiocatheter loaded with a compressed SEMS was inserted into the puncture site, and the stent was deployed at the RF ablation lesion by retracting the sheath while the pusher was held in place (Figure S2c). Cholangiography using a fluoroscopy system (MeteoR; NanoFocusRay Co., Iksan, Korea) was performed immediately after stent placement to evaluate stent patency and detect possible complications. The incision site was sequentially closed with sutures. Antibiotics (gentamicin, 80 mg/2 mL; SHIN POONG PHARM Ltd., Seoul, Korea) and analgesics (Keromin, ketorolac 30 mg; HANA PHARM Ltd., Seoul, Korea) were routinely administered intramuscularly for 3 days after the surgical procedure. All rabbits were euthanized by intravenous injection of potassium chloride at 4 weeks after the procedure. Body weight was measured weekly until sacrifice.

### 2.8. Cholangiographic Examination

Follow-up cholangiography was performed immediately before sacrifice in all rabbits to check the stent position and patency using a contrast medium (Telebrix Gastro; Guerbet, Villepinte, France). After a midline incision was made, the peritoneal adhesion was gently dissected, and cholangiography was performed by inserting the 18 G angiocatheter to the orifice of the ampulla of Vater in the same way as SEMS insertion. The luminal diameter of the stented extrahepatic bile duct was measured using the Radiant software (version 2020.2; Medixant, Poznan, Poland). Analysis of the cholangiography findings was performed in accordance with the agreement of three observers blinded to the group assignment.

### 2.9. Hematology

Venous blood samples (3 mL) were collected from each rabbit before sacrifice. The separated serum was stored at −20 °C until analysis. Aspartate transaminase (AST), alanine aminotransferase (ALT), alkaline phosphatase (ALP), gamma (γ)-glutamyl transferase (GGT), and total bilirubin levels were analyzed to evaluate hepatobiliary functions in all rabbits after the procedure.

### 2.10. Gross and Histological Examination

The biliary duct was surgically extracted. The degrees of biliary sludge and granulation tissue formation were evaluated by gross examination. Tissue samples were fixed in 10% neutral buffered formalin for 24 h. The stented bile duct was transversely sectioned at the proximal, middle, and distal parts. The cut stent wires and biliary sludge were carefully removed from the specimens and then embedded in paraffin. The slides were stained with hematoxylin-eosin (H&E) and Masson’s trichrome (MT). The bile duct cross-sections were stained with H&E to determine the degree of submucosal inflammatory cell infiltration, thickness of submucosal fibrosis, and percentage of granulation tissue in the stenotic area. The percentage was obtained using the Equation below.

$$100\times \left(1-\frac{\mathrm{stenotic}\;\mathrm{stented}\;\mathrm{area}}{\mathrm{original}\;\mathrm{stented}\;\mathrm{area}}\right)$$

The degree of inflammatory cell infiltration was subjectively classified into 1—mild, 2—mild to moderate, 3—moderate, 4—moderate to severe, and 5—severe, according to the distribution of inflammatory cells. The thickness of submucosal fibrosis and the degree of inflammatory cell infiltration were calculated as the average of eight values around the circumference. The MT-stained sections were performed to determine the degree of collagen deposition. The degree of collagen deposition was subjectively scored as 1—mild, 2—mild to moderate, 3—moderate, 4—moderate to severe, and 5—severe [10]. Histological results were visualized and analyzed using CaseViewer (3D HISTECH, Budapest, Hungary). Analyses of the histological findings were based on the consensus of three observers who were blinded to the experimental groups.

### 2.11. Immunohistochemistry Analysis

IHC analysis was performed on paraffin-embedded sections using terminal deoxynucleotidyl transferase-mediated dUTP nick and labeling (TUNEL; Millipore, Burlington, MA, USA), heat shock protein 70 (HSP 70; LS Bio, Seattle, WA, USA), and α-smooth muscle actin (α-SMA; LS Bio). The sections were visualized using a digital microscope viewer (CaseViewer). The degrees of TUNEL-, HSP 70-, and α-SMA-positive areas were subjectively determined using the following scores: 1—mild, 2—mild to moderate, 3—moderate, 4—moderate to severe, and 5—severe. IHC findings were also based on the consensus of three observers who were blinded to the experimental groups.

### 2.12. Statistical Analysis

Data were expressed as mean ± standard deviation (SD). Differences between the groups were analyzed using two-sample t test and Mann–Whitney U test, as appropriate. A *p* value of < 0.05 was considered statistically significant. Statistical analyses were performed using the SPSS software (version 27; IBM, Chicago, IL, USA).

## 3. Results

### 3.1. Surface Characteristics of AgNPs-Coated SEMS

**Figure 2:**
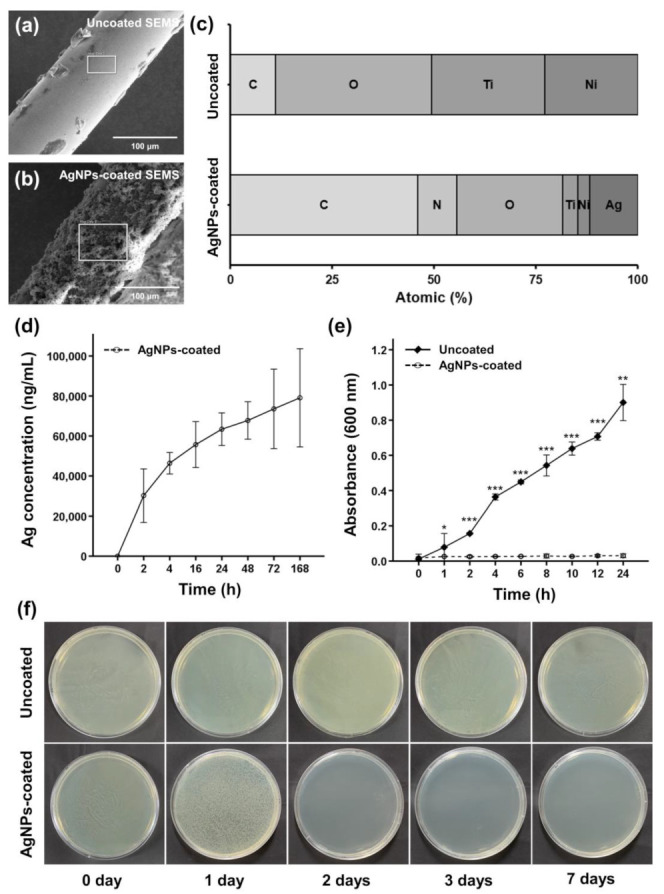
Surface and in vitro antibacterial characteristics of the uncoated and AgNPs-coated SEMSs. Scanning electron microscopy images of the (**a**) uncoated SEMS and (**b**) AgNPs-coated SEMS. (**c**) Elemental analysis through energy-dispersive X-ray spectroscopy confirmed that Ag was not present in the uncoated SEMS and was present in the AgNPs-coated SEMS. (**d**) Ag ions were rapidly released in the initial 2 h, and the amount of Ag ions released then gradually decreased. Bacterial activity at (**e**) 24 h and (**f**) 7 days showed that AgNPs-coated SEMS significantly inhibited bacterial growth compared to uncoated SEMS; * *p* < 0.05; ** *p* < 0.01; *** *p* < 0.001.

AgNPs-coated SEMSs were successfully fabricated through a two-step simple synthesis process. The surface of the uncoated samples was smooth and flawless, whereas that of the AgNPs-coated samples was rough, with AgNPs adhered. Elemental analysis through energy-dispersive X-ray spectroscopy revealed that the atomic percentage of Ag was 0% in uncoated SEMS and 11.8% in AgNPs-coated SEMS (Figure 2a–c).

### 3.2. Ag Ions Release Behavior of AgNPs-Coated SEMS

ICP-MS was used to measure the amount of AgNPs or Ag ions released by AgNPs-coated SEMS. Because no additional purification was performed, the released AgNPs and Ag ions could not be distinguished, and Figure 2d depicts the total released Ag ions by dissolving AgNPs in nitric acid. The release behavior of Ag ions from AgNPs-coated SEMS was rapid during the first 2 h, and then gradually decreased between 2 and 168 h, as shown in Figure 2d. The release amount of Ag ions was 30,201.5 ± 4382.5 ng/mL from 0 to 2 h, 16,202.8 ± 6111.4 ng/mL from 2 to 4 h, 9302.5 ± 2018.8 ng/mL from 4 to 16 h, 7692.2 ± 1499.8 ng/mL from 16 to 24 h, 4369.7 ± 414.4 ng/mL from 24 to 48 h, 5802.9 ± 5025.7 ng/mL from 48 to 72 h, and 5533.3 ± 2203. 8 ng/mL from 72 to 168 h.

### 3.3. In Vitro Antibacterial Efficacy of AgNPs-Coated SEMS

The antibacterial activity of uncoated and AgNPs-coated SEMSs over 24 h is shown in Figure 2e. The OD_600_ value of *E. coli* under treatment with AgNPs-coated SEMS (0.03 ± 0.01) was significantly different from that under treatment with uncoated SEMS (0.08 ± 0.03) at the 1 h time point (*p* < 0.05). Afterward, the *E. coli* suspension cultured with AgNPs-coated SEMS mostly maintained the OD_600_ value. Based on the OD_600_ values, it was confirmed that the bacteria grew significantly and continuously under culture with the uncoated SEMS for 24 h, compared to that under culture with the AgNPs-coated SEMS (*p* < 0.001 in all conditions except at 24 h, in which the *p* value was <0.01, two-sample *t*-test).

Bacterial growth on LB agar was imaged for 7 days, and the results are shown in Figure 2f. A clear zone indicated inhibited bacterial growth. The medium in the uncoated SEMS group presented no clear zone. After 1 day of incubation, bacterial growth in the AgNPs-coated SEMS group was smaller than that in the uncoated SEMS group.

### 3.4. Protocol for RF Ablation in the Rabbit Bile Duct

**Figure 3:**
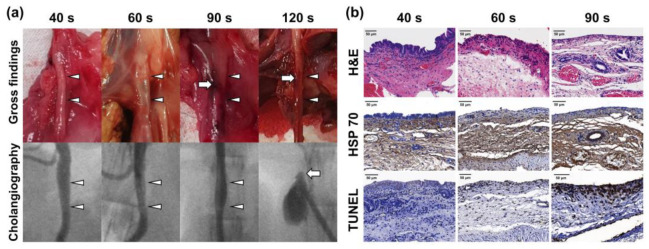
Gross, cholangiography, histological, and immunohistochemistry findings immediately after RF ablation for 40, 60, 90, and 120 s at 70 °C. (**a**) Gross and cholangiography findings confirmed that the damage in the bile duct worsened over time. (**b**) As the duration of ablation was increased, submucosal thickness decreased, thermal damage worsened, and cell death increased.

RF ablation using a modified RF electrode was successfully performed in all rabbits. After 40 s of ablation, no mucosal injury was found in the bile duct. However, mucosal injuries and thinning of the bile duct were observed after ablation for 60 s. After 90 s of ablation, the bile duct was tanned and presented severe mucosal injuries. Ductal perforation was observed in the bile duct ablated for 120 s. For this reason, histological analysis could not be performed. Regarding the cholangiographic findings, newly developed stricture formation was not observed after RF ablation for 40, 60, and 90 s. Leakage of the contrast medium through the perforated bile duct was detected in the bile duct ablated for 120 s (Figure 3a).

Submucosal thickness in the ablated bile duct gradually decreased in a time-dependent manner. The epithelium was observed in the bile duct ablated for 40 s, but not in the bile duct ablated for 60 and 90 s. HSP 70-positive areas were observed in all rabbits. The TUNEL-positive area gradually increased in a time-dependent manner (Figure 3b).

### 3.5. Procedural Outcomes in the In Vivo Study

**Figure 4:**
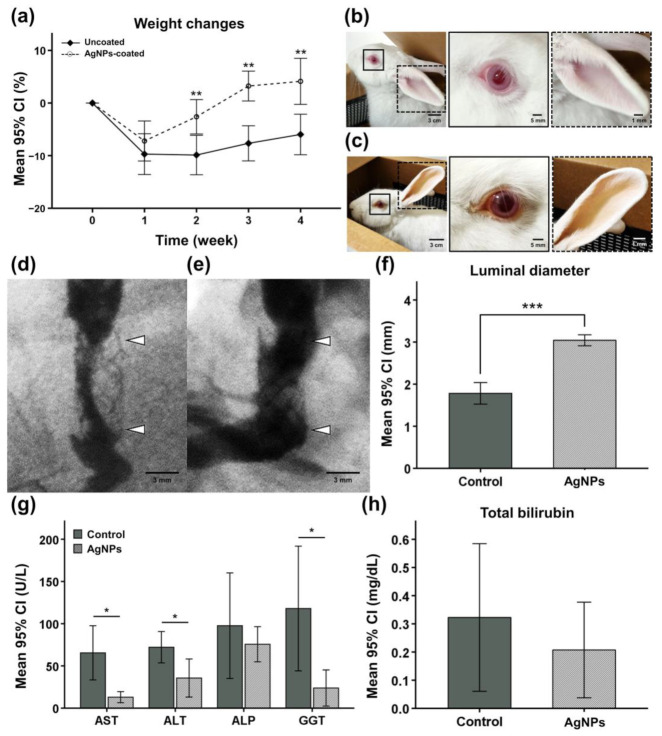
Procedural outcomes, cholangiography results, and hematological findings at 4 weeks after RF ablation with stent placement. (**a**) All rabbits recovered the weight loss caused by the procedure, and the recovery rate of the AgNPs group was significantly higher than of the control group. Compared with (**b**) normal rabbits, rabbits with (**c**) jaundice had yellow eyes (solid line square, magnification ×4.3) and ears (dashed line square, magnification ×23.1), and jaundice occurred only in the control group. The (**d**) uncoated SEMS (arrowheads) after RF ablation had a lower patency than the (**e**) AgNPs-coated SEMS (arrowheads). (**f**) Mean luminal diameter of the stented extrahepatic bile duct. (**g**) Mean levels of AST, ALT, ALP, GGT, and (**h**) total bilirubin in the two groups. CI, confidence interval; AST, aspartate transaminase; ALT, alanine aminotransferase; ALP, alkaline phosphatase; GGT, gamma (γ)-glutamyl transferase; * *p* < 0.05, ** *p* < 0.01, *** *p* < 0.001.

Stent placement immediately after RF ablation using a modified RF electrode was successfully performed without procedure-related complications in all rabbits. Body weight decreased after the procedure in the two groups, but all rabbits gradually recovered their weight. The mean weight change ratio before the procedure and before sacrifice between the groups was significantly larger in the AgNPs group (4.12 ± 2.75%) than in the control group (−5.97 ± 3.11%, *p* < 0.01, two-sample *t* test) (Figure 4a). Additionally, jaundice was observed in 3 out of 6 (50%) rabbits in the control group (Figure 4c).

### 3.6. Cholangiographic Findings

All rabbits successfully underwent follow-up cholangiography at 4 weeks after the procedure. The extrahepatic bile duct was dilated in the proximal and distal portion from the stented bile duct owing to severe in-stent stenosis in the control group. Total obstruction of the stent was observed in two rabbits of the control group. In the AgNP group, the stent patency was relatively good, and no rabbits presented jaundice symptoms (Figure 4d,e). The mean (±SD) luminal diameter in the control group (3.04 ± 0.51 mm) was greater than that in the AgNPs group (1.78 ± 0.53 mm, *p* < 0.001, Mann–Whitney U test) (Figure 4f).

### 3.7. Hematology

The mean (±SD) AST (65.5 ± 30.6 U/L vs. 13.0 ± 2.6 U/L, *p* < 0.01), ALT (72.2 ± 17.7 U/L vs. 35.7 ± 9.1 U/L, *p* < 0.05), and GGT (118.0 ± 70.4 U/L vs. 23.8 ± 8.6 U/L, *p* < 0.05) levels were significantly higher in the control group than in the AgNPs group. The mean (±SD) ALP (97.9 ± 59.6 U/L vs. 75.7 ± 8.4 U/L, *p* = 0.413) and total bilirubin (0.3 ± 0.2 mg/dL vs. 0.2 ± 0.1 mg/dL, *p* = 0.350) levels were not significantly different between the two groups (Figure 4g,h).

### 3.8. Gross and Histological Findings

**Figure 5:**
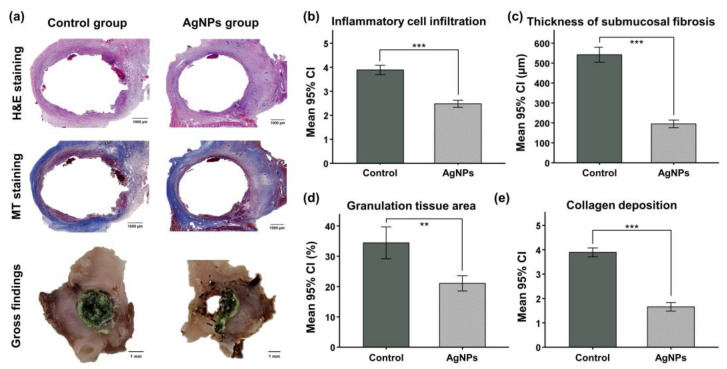
Gross and histological findings. (**a**) Representative images of gross and histologic findings revealed that the degree of tissue hyperplasia was higher in the control group than in the AgNPs group. (**b**–**e**) Histological results of the stented extrahepatic bile duct in the two groups. CI, confidence interval; ** *p* < 0.01, *** *p* < 0.001.

In the gross findings, sludge formation into the stented bile duct was more pronounced in the control group than in the AgNPs group. However, biofilm formation adjacent to the stent was found in all groups. The mean (±SD) degree of inflammatory cell infiltration was significantly higher in the control group (3.89 ± 0.93) than in the AgNPs group (2.48 ± 0.63, *p* < 0.001). The mean (±SD) thickness of submucosal fibrosis was significantly larger in the control group (542.0 ± 164.7 μm) than in the AgNPs group (195.2 ± 83.5 μm, *p* < 0.001). The mean (±SD) percentage of granulation tissue area (34.4 ± 6.9% vs. 21.1 ± 2.4%, *p* < 0.01) and the degree of collagen deposition (3.89 ± 0.97 vs. 1.66 ± 0.78, *p* < 0.001) were also significantly higher in the control group than in the AgNPs group (Figure 5).

### 3.9. Immunohistochemistry Findings

**Figure 6:**
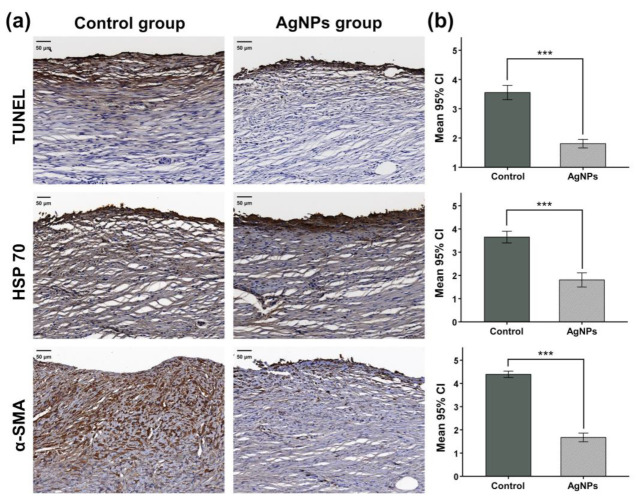
Representative microscopic IHC staining images and findings. (**a**) Representative images of terminal deoxynucleotidyl transferase-mediated dUTP nick and labeling (TUNEL)-, heat shock protein 70 (HSP 70)-, and α-smooth muscle actin (α-SMA)-stained sections are shown. (**b**) The mean degrees of positive staining were significantly higher in the control group than in the AgNPs group. CI, confidence interval; *** *p* < 0.001.

The IHC findings are shown in Figure 6. The mean (±SD) degrees of TUNEL-positive staining were significantly higher in the control group (3.55 ± 1.18) than in the AgNPs group (1.81 ± 0.73, *p* < 0.001). The mean (±SD) degrees of positive staining for HSP 70 (3.65 ± 1.00 vs. 1.81 ± 0.83, *p* < 0.001) and α-SMA (4.39 ± 0.80 vs. 1.67 ± 0.89, *p* < 0.001) were also significantly higher in the control group than in the AgNPs group.

## 4. Discussion

The results of our study showed that AgNPs successfully suppressed the bacterial and inflammatory responses caused by heat and stents. The number of *E. coli* was significantly lower after culture with the AgNPs-coated SEMS than after culture with the uncoated SEMS. Moreover, the AgNPs group showed better patency than the control group. These findings suggest that AgNPs inhibits the formation of bacterial biofilm through an antibacterial effect. Although the antibacterial activity of AgNPs is not entirely understood, previous studies have found that AgNPs can continually release Ag ions and accumulate in microorganisms, generating reactive oxygen species and killing microbes [34,35,36]. Additionally, the AgNPs can accumulate in the bacterial membrane, denature the cell membrane, and result in the bacteria’s death [37,38].

In addition, the degree of inflammatory cell infiltration, thickness of submucosa fibrosis, granulation tissue area, and collagen deposition were significantly lower in the AgNPs group. IHC results also confirmed that the proportions of TUNEL-, HSP 70-, and α-SMA-positive cells were all significantly lower in the AgNPs group. These findings indicate that AgNPs promote injury recovery by inhibiting bacterial and inflammatory responses.

We successfully coated SEMS with AgNPs using previously published methods [10]. In vitro release behavior tests in PBS confirmed that AgNPs release was rapid in the initial 7 days and gradually decreased over time. The antibacterial effect of AgNPs within 1 day was assessed by measuring the OD_600_ value of *E. coli* suspension cultured with uncoated and coated stents [39]. Bacterial activity in the solid agar medium was examined after 7 days of incubation, and the clear zone suggested that bacterial growth was inhibited [40,41]. Moreover, bacterial growth was significantly lower after culture with AgNPs-coated SEMS for 1 day than after culture with the uncoated SEMS. Bacterial activity assays for 7 days revealed that under incubation with the AgNPs-coated SEMS, bacterial growth was gradually suppressed since the first day, and a clear zone formed since the second day onward. Our results showed that the AgNPs-coated SEMS effectively inhibited the growth of *E. coli* through steady release for 7 days. Nevertheless, these findings should be verified in future studies with longer follow-up times to confirm the long-term efficacy of AgNPs.

The human common bile duct and the rabbit bile duct differ in their characteristics, such as the submucosal thickness. Therefore, we first identified the appropriate ablation time for the rabbit common bile duct instead of using the clinically used conditions. RF ablation was performed for 40, 60, 90, and 120 s. After ablation for 120 s, the perforation was immediately observed, and thus further examination was not performed. Gross examination and cholangiography confirmed that the damage to the bile duct increased as the ablation duration was prolonged. Non-vascular luminal organs, such as the bile duct, have a thin submucosa. Therefore, an appropriate duration is required for RF ablation of the rabbit common bile duct. After ablation for 40 s, the submucosal thickness was almost similar to that of the normal bile duct. After ablation for 60 and 90 s, the thickness decreased by approximately 42.8% and 26.3%, respectively, compared to that in the normal bile duct. Heat damage was confirmed in all conditions, and the degree of cell death was the greatest after 90 s of ablation. Based on the results of this experiment, the optimal ablation condition for the rabbit bile duct was set as 60 s.

After the procedure, all rabbits showed rapid weight loss, which then gradually recovered. However, the recovery rate of the control group was lower than that of the AgNPs group. In addition, jaundice was observed in 50% of the rabbits in the control group. Jaundice is mainly caused by cholestasis [42]. Thus, we predicted the development of cholestasis in rabbits of the control group. All rabbits underwent cholangiography prior to euthanasia. The patency was significantly higher in the AgNPs group than in control group, and the diameter of the bile duct outside the stent was enlarged in the control group. These findings suggested that cholestasis occurred owing to tissue hyperplasia inside the bile duct where the stent was placed. Biliary fluid accumulation due to tissue hyperplasia in the bile ducts is predicted to cause excessive pressure within the bile ducts, thereby increasing the volume of the bile ducts [43]. In addition, the results of hematology assay demonstrated higher AST, ALT, ALP, GGT, and total bilirubin levels of the control group than the AgNPs group. As the most accurate predictors of bile duct obstruction and inflammation, the increases in ALP, GGT, and bilirubin levels suggest hepatic dysfunction, bile duct obstruction, and inflammation of the bile duct in the control group [44,45,46,47]. In this study, a wide range of the ALP, GGT, and total bilirubin levels were observed, and the levels were particularly higher in the control group where jaundice was noticed. This may be explained by the small number of rabbits in each group, given that wide range of biochemical levels is observed when bile duct obstruction occurs [48]. Therefore, these results indicate that AgNPs contribute to inhibiting tissue hyperplasia.

In vivo parameters were evaluated through gross examination, histological evaluation, and IHC analysis. Visually, the bile duct of the control group was filled with sludge and biofilm. The degree of inflammatory cell infiltration, thickness of submucosal fibrosis, area of granulation tissue, and degree of collagen deposition were all significantly lower in the AgNPs group than in the control group. Positivity for TUNEL, HSP 70, and α-SMA was also significantly lower in the AgNPs group than in the control group. These findings implied that AgNPs promoted the recovery of thermal and mechanical damage caused by RF ablation and stent placement. RF ablation and stent placement are susceptible to bacterial infection and inflammatory responses. Infection with and inflammatory responses to microorganisms, such as *E. coli*, are the main causes of delayed injury healing [49,50,51]. Consequently, bacterial infection and inflammatory response delayed injury healing as well as increased the risk of restenosis and sepsis through repeated infection and inflammation. Therefore, we propose that AgNPs promote the healing of heat-induced injury and mechanical damage by inhibiting bacterial infection and inflammatory responses.

Our study, however, had several limitations. First, the experiment was conducted in the normal bile duct without malignant obstruction. The injury healing pattern and experimental conditions after injury due to RF ablation and stent placement may be different from those of a stenosis model. Second, the period for AgNPs release and bacterial activity evaluation was insufficient. It is necessary to investigate whether AgNPs are continuously released and bacterial activity is inhibited during the stent placement period required in clinical practice. Additionally, evidence of AgNPs’ action on *Staphylococcus* spp., pathogen responsible for biofilm formation and cholangitis, is necessary later. Nevertheless, our findings support the basic concept that the AgNPs-coated SEMS inhibits tissue hyperplasia and bacterial growth induced by stent placement immediately after RF ablation in the rabbit bile duct.

## 5. Conclusions

Minimally invasive management of MBO using intraductal RF ablation with stent technology is currently limited by the development of tissue hyperplasia and bacterial infection adjacent to the SEMS. We were able to successfully coat SEMSs with AgNPs to suppress the inflammatory and bacterial response caused by stent placement immediately after RF ablation. The AgNPs-coated SEMS significantly inhibited tissue hyperplasia and biofilm induced by heat and mechanical damage in the rabbit common bile duct. Although further preclinical studies are required to investigate the efficacy and safety of the AgNPs-coated SEMS, the developed method of RF ablation with AgNPs-coated SEMS should be a promising therapeutic strategy for the prevention of RF ablation- and stent-related complications in patients with unresectable MBO.

## Data Availability

Not applicable.

## Supplementary Materials

The following supporting information can be downloaded at: https://www.mdpi.com/article/10.3390/pharmaceutics14020412/s1, Figure S1: Radiofrequency (RF) electrode (RF Electrode F180707; APRO KOREA, Gunpo, Korea) used to ablate the rabbit extrahepatic common bile duct. (a) Overall image of the commercialized RF electrode. Magnified image of (b) the needle with the existing sharp point and (c) the needle with a tapered shape following grinding. Figure S2: Technical steps involved in stent placement immediately after radiofrequency (RF) ablation. (a) The ampulla of Vater (white star) was exposed and punctured, and (b) the middle of the RF electrode (arrowheads) was placed 2.5 cm from the puncture site. (c) The self-expandable metal stents (SEMSs; arrowheads) were placed in the RF lesion.
