# Supplementary material for: Silver Nanofunctionalized Stent after Radiofrequency Ablation Suppresses Tissue Hyperplasia and Bacterial Growth
Source: Pharmaceutics. 2022 Feb 14;14(2):412. doi: 10.3390/pharmaceutics14020412 (PMC8876892; doi:10.3390/pharmaceutics14020412)
Supplement: Supplementary file 1 [file pharmaceutics-14-00412-s001.zip › pharmaceutics-1584522-supplementary.pdf]

# Supplementary materials: Silver Nanofunctionalized Stent after Radiofrequency Ablation Suppresses Tissue Hyperplasia and Bacterial Growth

**Yubeen Park, Dong-Sung Won, Ga-Hyun Bae, Dae Sung Ryu, Jeon Min Kang, Ji Won Kim, Song Hee Kim, Chu Hui Zeng, Wooram Park, Sang Soo Lee and Jung-Hoon Park**

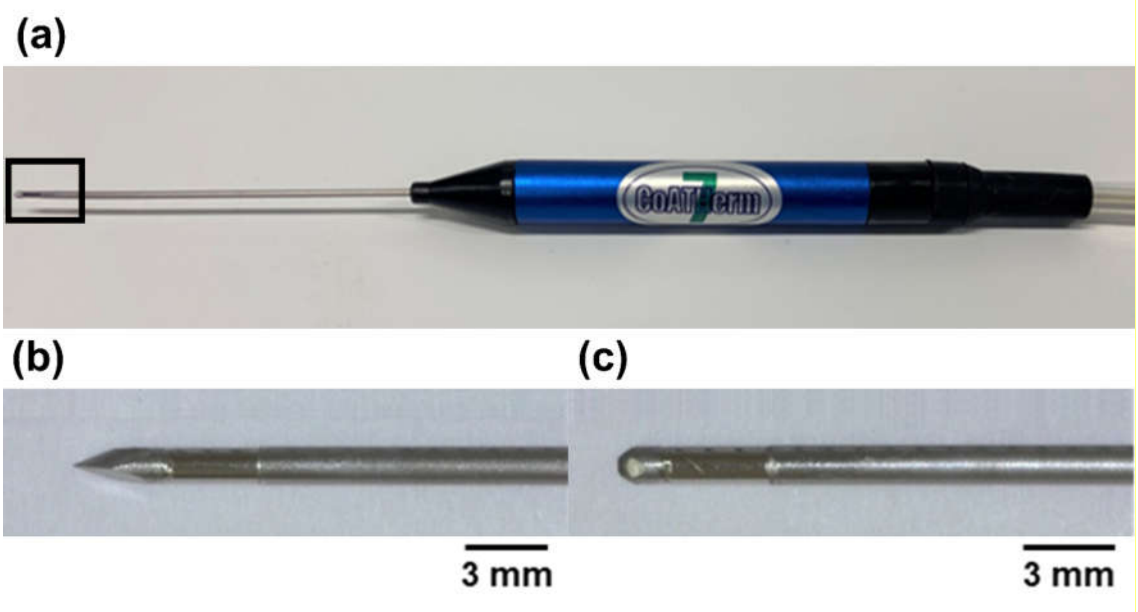


**Figure S1.** Radiofrequency (RF) electrode (RF Electrode F180707; APRO KOREA, Gunpo, Korea) used to ablate the rabbit extrahepatic common bile duct. (**a**) Overall image of the commercialized RF electrode. Magnified image of (**b**) the needle with the existing sharp point and (**c**) the needle with a tapered shape following grinding.

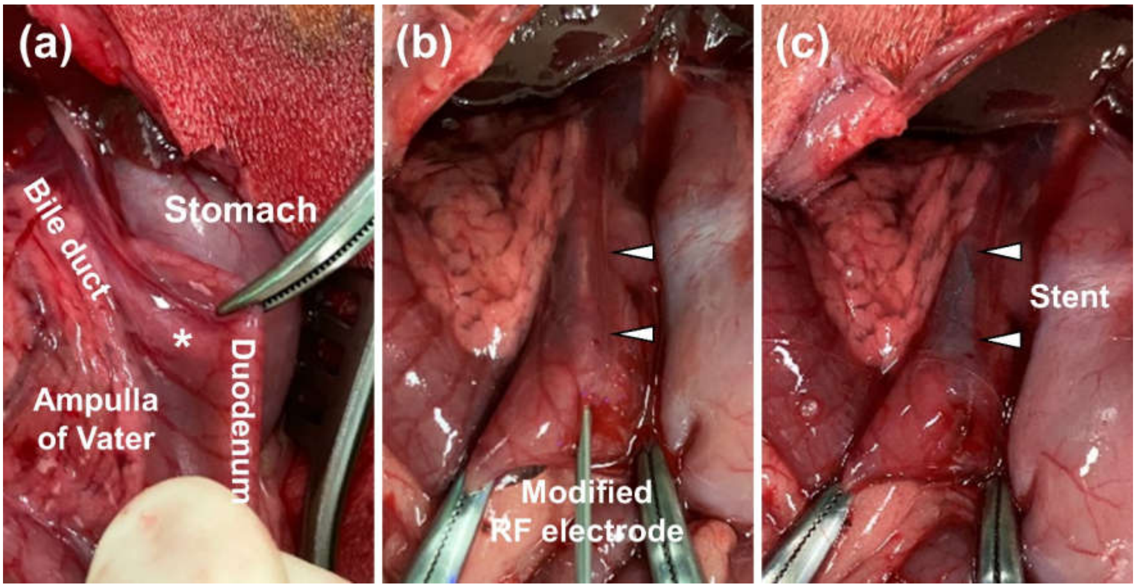


**Figure S2.** Technical steps involved in stent placement immediately after radiofrequency (RF) ablation. (**a**) The ampulla of Vater (white star) was exposed and punctured, and (**b**) the middle of the RF electrode (arrowheads) was placed 2.5 cm from the puncture site. (**c**) The self-expandable metal stents (SEMSs; arrowheads) were placed in the RF lesion.
